# Author Correction: Rad52’s DNA annealing activity drives template switching associated with restarted DNA replication

**DOI:** 10.1038/s41467-023-37201-9

**Published:** 2023-03-14

**Authors:** Anastasiya Kishkevich, Sanjeeta Tamang, Michael O. Nguyen, Judith Oehler, Elena Bulmaga, Christos Andreadis, Carl A. Morrow, Manisha Jalan, Fekret Osman, Matthew C. Whitby

**Affiliations:** Department of Biochemistry, University of Oxford, South Parks Road, Oxford, OX1 3QU UK

**Keywords:** DNA recombination, Genomic instability

Correction to: *Nature Communications*; 10.1038/s41467-022-35060-4;published online 26 November 2022

The original version of this Article omitted from the author list the author Manisha Jalan, who is from the Department of Biochemistry, University of Oxford. Consequently, the following was added to the Author Contributions: A.K., S.T., M.O.N., J.O., and M.J.: conceived and performed experiments. E.B. and C.A. performed experiments; C.A.M. and F.O. provided reagents, expertise, and feedback; M.C.W. conceived experiments, wrote the paper, and secured funding’. This has been corrected in both the PDF and HTML versions of the Article.

