# Supplementary material for: Rad52’s DNA annealing activity drives template switching associated with restarted DNA replication
Source: Nat Commun. 2022 Nov 26;13:7293. doi: 10.1038/s41467-022-35060-4 (PMC9701231; doi:10.1038/s41467-022-35060-4)
Supplement: Supplementary file 1 — Supplementary Information [file 41467_2022_35060_MOESM1_ESM.pdf]

Rad52's DNA annealing activity drives template switching associated with restarted DNA replication

Anastasiya Kishkevich, Sanjeeta Tamang, Michael O. Nguyen, Judith Oehler, Elena Bulmaga, Christos Andreadis, Carl A. Morrow, Fekret Osman and Matthew C. Whitby

Department of Biochemistry, University of Oxford, South Parks Road, Oxford, OX1 3QU UK

SUPPLEMENTARY INFORMATION

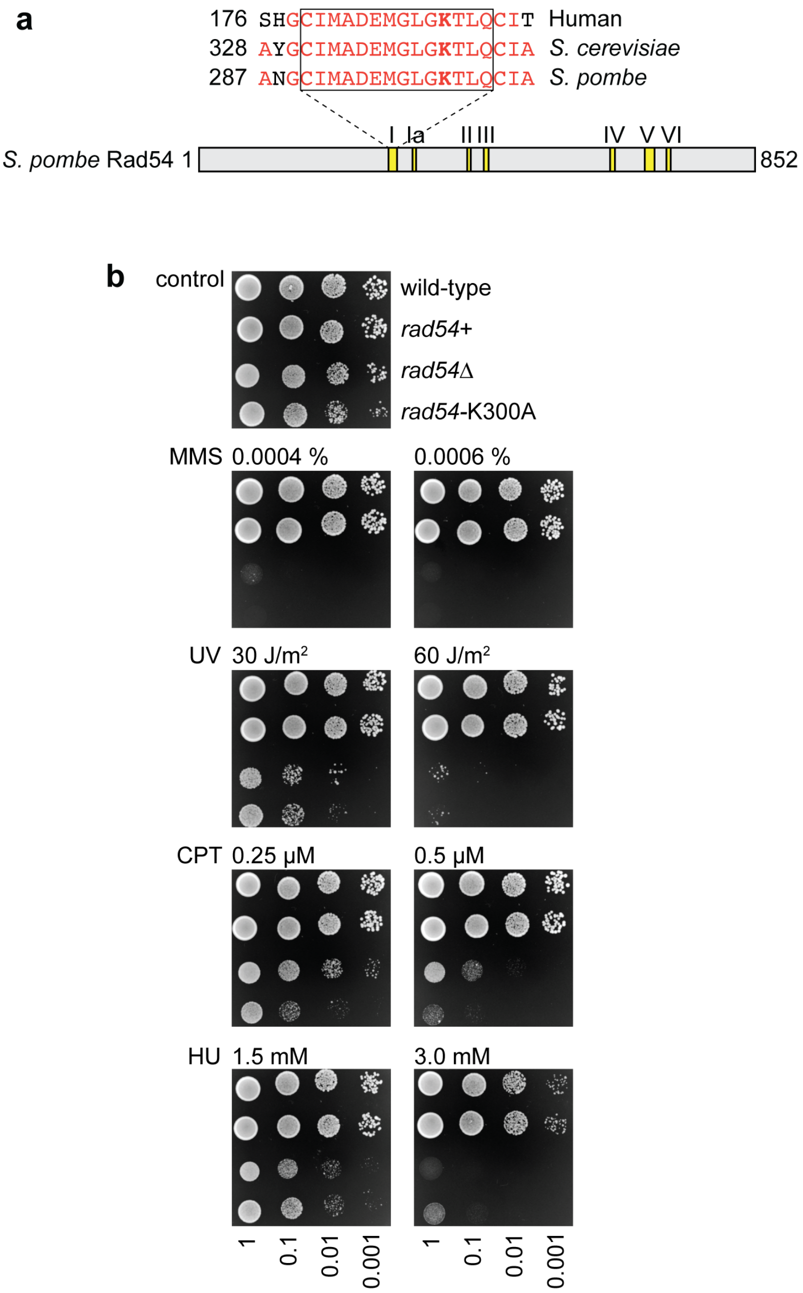

Supplementary Figure 1. A *rad54*-K300A mutant exhibits a similar sensitivity to genotoxins as a *rad54*Δ mutant

a Schematic of Rad54 showing the seven conserved helicase/motor motifs (in yellow) and highlighting conserved lysine 300 in motif I. **b** Spot assay comparing the genotoxin sensitivities of the fission yeast strains MCW1221 (wild-type), MCW6910 (*rad54*$^{+}$-*natMX4*), MCW1230 (*rad54*Δ) and MCW6911 (*rad54*-K300A-*natMX4*). The genotoxins are: methyl methanesulfonate (MMS); ultraviolet light (UV); camptothecin (CPT); and hydroxyurea (HU).

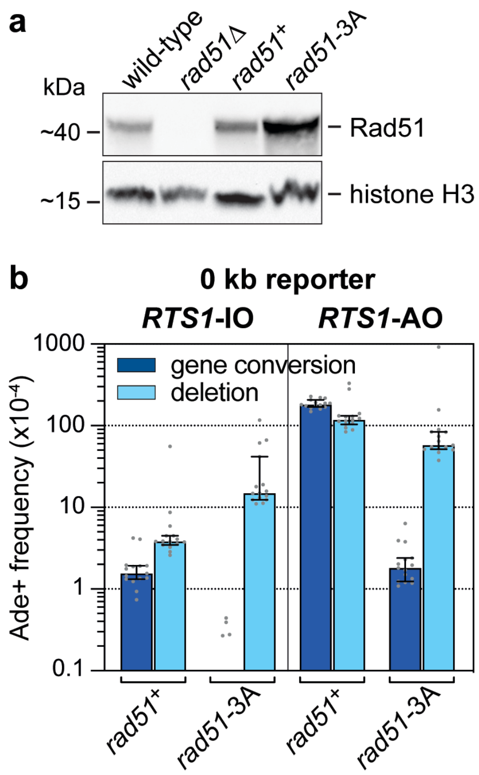

Supplementary Figure 2. Spontaneous and *RTS1*-AO-induced recombination in a *rad51*-3A mutant

a Western blot showing Rad51 in whole cell extracts from wild-type (MCW8888), *rad51*Δ (MCW9002), *rad51*$^{+}$-*kanMX6* (MCW9856) and *rad51*-3A-*kanMX6* (MCW9851) strains. Histone H3 serves as a loading control. An independent repeat of this experiment gave similar results. **b** Frequency of spontaneous (*RTS1*-IO) and *RTS1*-AO-induced Ade^{+} recombinants in *rad51*$^{+}$ and *rad51*-3A strains with the 0 kb reporter. Data are presented as median values ± interquartile range with individual data points shown as grey dots. The data are also reported in Supplementary Table 1, which includes the strain numbers, the number of colonies tested for each strain (*n*) and p-values. Further details of the statistical analysis are reported in Supplementary Data 1. Source data are provided as a Source Data file.

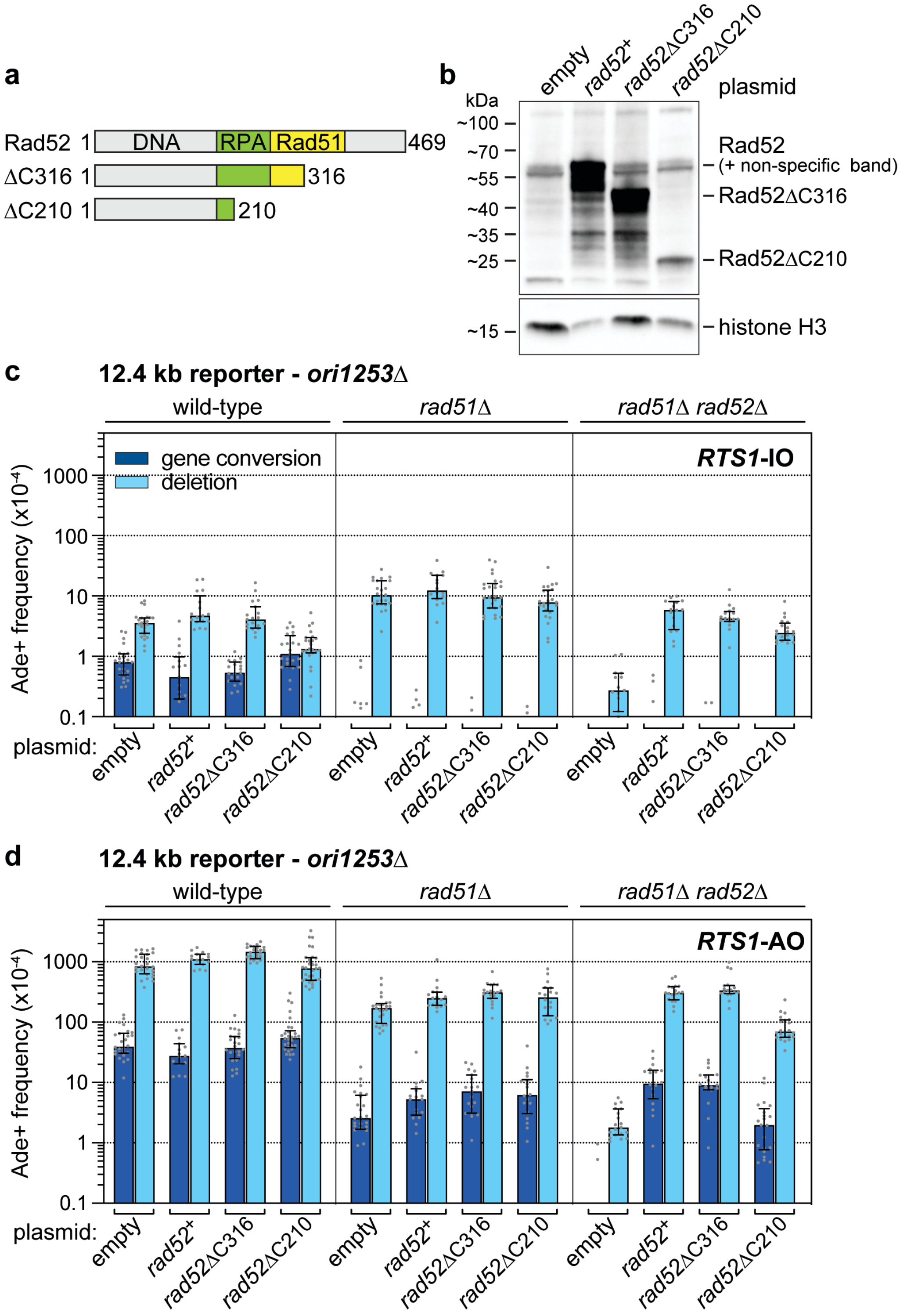

Supplementary Figure 3. Rad52's N-terminal domain is sufficient to promote Rad51-independent RDR-associated template switching when over-expressed

a Schematic showing the two Rad52 C-terminal truncations, ΔC316 and ΔC210. **b** Western blot showing the relative amounts of full length and truncated Rad52 in whole cell extracts from a wild-type strain (MCW8888) carrying plasmid as indicated.

Histone H3 serves as a loading control. An independent repeat of this experiment gave similar results. **c** Frequency of spontaneous (*RTS1*-IO) Ade$^+$ recombinants in wild-type and mutant strains with *ori1253*Δ and the 12.4 kb reporter, and containing plasmids as indicated. **d** Frequency of *RTS1*-AO-induced Ade$^+$ recombinants in wild-type and mutant strains with *ori-1253*Δ and the 12.4 kb reporter, and containing plasmids as indicated. **c-d** Data are presented as median values ± interquartile range with individual data points shown as grey dots. The data are also reported in Supplementary Table 1, which includes the strain numbers, the number of colonies tested for each strain (*n*) and p-values. Further details of the statistical analysis are reported in Supplementary Data 1. Source data are provided as a Source Data file.

Supplementary Table 1: Recombination frequencies

Relevant Genotype and Strain no.	*RTS1* presence and orientation	Recomb-ination reporter[a]	Number of colonies tested (*n*)	Ade+ His+ recombinant frequency (x 10^{-4})		Ade+ His- recombinant frequency (x 10^{-4})		Figure(s)
				Median (95% CI)[b]	p-value[c]	Median (95% CI)[b]	p-value[c]	
wild-type MCW4712	IO	0 kb reporter	99	1.33 (1.16 –1.47)	-	3.49 (3.23 – 3.64)	-	2a
wild-type MCW4713	AO	0 kb reporter	133	128.0 (124.3 – 132.4)	-	86.8 (84.6 – 96.3)	-	2b
*rad51*Δ MCW1691	IO	0 kb reporter	59	0	<0.0001[d]	16.7 (15.1 – 19.4)	<0.0001[d]	2a
*rad51*Δ MCW1692	AO	0 kb reporter	69	1.32 (0.81 – 1.82)	<0.0001[e]	72.3 (60.6 – 82.9)	0.0802[e]	2b
*rad51*Δ *rad52*Δ MCW1695	IO	0 kb reporter	11	0	<0.0001[d]	3.43 (2.87 – 5.20)	>0.9999[d]	2a
*rad51*Δ *rad52*Δ MCW1696	AO	0 kb reporter	15	0.02 (0.00 – 0.07)	<0.0001[e]	4.23 (3.99 – 5.54)	<0.0001[e]	2b
rad52-R45A MCW9607	IO	0 kb reporter	22	0.94 (0.79 – 1.09)	>0.9999[d]	3.86 (3.28 – 4.27)	>0.9999[d]	2a
rad52-R45A MCW9608	AO	0 kb reporter	26	99.5 (76.29 – 124.3)	>0.9999[e]	82.03 (65.61 – 114.6)	>0.9999[e]	2b
*rad51*Δ *rad52*-R45A MCW9723	IO	0 kb reporter	30	0	<0.0001[d]	2.93 (2.62 – 3.99)	>0.9999[d]	2a
*rad51*Δ *rad52*-R45A MCW9725	AO	0 kb reporter	29	0	<0.0001[e]	5.44 (4.11 – 7.11)	<0.0001[e]	2b
*rad54*Δ MCW2651	IO	0 kb reporter	20	0	<0.0001[d]	5.24 (4.31 – 6.79)	0.2026[d]	2a
*rad54*Δ MCW8392	AO	0 kb reporter	16	0.91 (0.53 – 1.27)	<0.0001[e] >0.9999[aa]	26.81 (20.90 – 52.07)	<0.0001[e]	2b
rad54-K300A MCW7034	IO	0 kb reporter	17	0.00 (0.00 – 0.12)	<0.0001[d]	12.73 (11.14 – 14.75)	<0.0001[d]	2a
rad54-K300A MCW7037	AO	0 kb reporter	18	0.39 (0.00 – 0.95)	<0.0001[e] >0.9999[aa]	29.31 (25.20 – 37.00)	<0.0001[e]	2b
*rad55*Δ MCW2650	IO	0 kb reporter	37	0 (0.00 – 0.13)	<0.0001[d]	11.73 (10.97 – 12.51)	<0.0001[d]	2a
*rad55*Δ MCW2655	AO	0 kb reporter	40	0.65 (0.53 – 0.96)	<0.0001[e] >0.9999[aa]	24.01 (20.00 – 27.69)	<0.0001[e]	2b
*rad57*Δ MCW6539	IO	0 kb reporter	19	0.1 (0.00 – 0.19)	<0.0001[d]	11.91 (9.33 – 15.05)	<0.0001[d]	2a
*rad57*Δ MCW6454	AO	0 kb reporter	19	0.44 (0.21 – 1.07)	<0.0001[e] >0.9999[aa]	38.42 (33.01 – 53.29)	<0.0001[e]	2b
*swi5*Δ MCW2647	IO	0 kb reporter	54	1.01 (0.91 – 1.17)	>0.9999[d]	6.4 (5.96 – 6.68)	<0.0001[d]	2a

*swi5*Δ MCW2653	AO	0 kb reporter	53	68.39 (61.11 – 78.57)	0.0430[e]	71.97 (64.49 – 80.35)	0.2087[e]	2b
*sfr1*Δ MCW6497	IO	0 kb reporter	25	0.81 (0.74 – 1.07)	0.8128[d]	5.25 (4.79 – 6.17)	0.1040[d]	2a
*sfr1*Δ MCW6499	AO	0 kb reporter	13	91.07 (76.44 – 103.1)	>0.9999[e]	106.3 (93.65 – 145.6)	>0.9999[e]	2b
*rdl1*Δ MCW6501	IO	0 kb reporter	36	0.67 (0.53 – 0.77)	0.0006[d]	6.16 (5.85 – 6.73)	0.0004[d]	2a
*rdl1*Δ MCW6468	AO	0 kb reporter	40	9.2 (8.15 – 10.43)	<0.0001[e]<br>0.0004[aa]	36.79 (31.65 – 42.71)	<0.0001[e]	2b
*rlp1*Δ MCW6505	IO	0 kb reporter	20	0.75 (0.65 – 0.91)	0.1382[d]	5.01 (4.28 – 5.33)	>0.9999[d]	2a
*rlp1*Δ MCW6507	AO	0 kb reporter	23	7.9 (6.59 – 8.96)	0.0001[e]<br>0.0154[aa]	28.9 (25.00 – 34.42)	<0.0001[e]	2b
*sws1*Δ MCW6503	IO	0 kb reporter	18	0.90 (0.71 – 1.25)	>0.9999[d]	2.84 (2.16 – 3.23)	>0.9999[d]	2a
*sws1*Δ MCW6470	AO	0 kb reporter	23	31.78 (29.77 – 37.43)	0.0173[e]<br><0.0001[aa]	23.24 (21.12 – 30.12)	<0.0001[e]	2b
*rad55*Δ *swi5*Δ MCW2912	IO	0 kb reporter	33	0	<0.0001[d]	20.11 (16.53 – 25.93)	<0.0001[d]	2a
*rad55*Δ *swi5*Δ MCW2916	AO	0 kb reporter	34	0.64 (0.00 – 1.05)	<0.0001[e]	47.25 (42.86 – 57.50)	<0.0001[e]	2b
*rad55*Δ *rdl1*Δ MCW6828	IO	0 kb reporter	30	0.10 (0.05 – 0.15)	<0.0001[d]	16.24 (14.05 – 18.27)	<0.0001[d]	2a
*rad55*Δ *rdl1*Δ MCW6832	AO	0 kb reporter	34	1.20 (0.77 – 1.65)	<0.0001[e]	42.40 (34.69 – 50.75)	<0.0001[e]	2b
*swi5*Δ *rdl1*Δ MCW7240	IO	0 kb reporter	33	0.72 (0.61 – 0.93)	0.0327[d]	11.73 (10.85 – 12.78)	<0.0001[d]	2a
*swi5*Δ *rdl1*Δ MCW7242	AO	0 kb reporter	32	5.58 (4.07 – 7.94)	<0.0001[e]	36.87 (33.96 – 41.79)	<0.0001[e]	2b
*rad55*Δ *swi5*Δ *rdl1*Δ MCW7250	IO	0 kb reporter	17	0.00 (0.00 – 0.15)	<0.0001[d]	22.91 (17.83 – 25.95)	<0.0001[d]	2a
*rad55*Δ *swi5*Δ *rdl1*Δ MCW7252	AO	0 kb reporter	17	0.93 (0.50 – 1.20)	<0.0001[e]	36.24 (32.67 – 40.00)	<0.0001[e]	2b
*rad51*Δ *rad55*Δ MCW6430	IO	0 kb reporter	19	0	<0.0001[d]	20.91 (13.01 – 29.17)	<0.0001[d]	2a
*rad51*Δ *rad55*Δ MCW6565	AO	0 kb reporter	20	1.75 (1.00 – 2.65)	<0.0001[e]<br>>0.9999[aa]	86.95 (64.77 – 108.7)	>0.9999[e]	2b
*rad51*Δ *swi5*Δ MCW6836	IO	0 kb reporter	20	0	<0.0001[d]	31.64 (25.53 – 38.04)	<0.0001[d]	2a

*rad51*Δ *swi5*Δ MCW6842	AO	0 kb reporter	19	1.82 (1.16 – 4.62)	<0.0001[e]	94.05 (83.67 – 148.1)	>0.9999[e]	2b
rad51+ MCW9928	IO	0 kb reporter	15	1.56 (1.32 – 1.93)	-	3.81 (3.47 – 4.50)	-	S2b
rad51+ MCW9931	AO	0 kb reporter	15	180.2 (169.8 – 207.2)	-	117.7 (103.8 – 132.2)	-	S2b
rad51-3A MCW9904	IO	0 kb reporter	15	0.059 (0.00 – 0.27)	<0.0001[ab]	14.94 (12.4 – 41.6)	<0.0001[ab]	S2b
rad51-3A MCW9906	AO	0 kb reporter	15	1.81 (1.24 – 2.40)	<0.0001[ac]	57.47 (51.5 – 84.0)	0.0004[ac]	S2b
wild-type MCW7257	IO	12.4 kb reporter	65	0.9 (0.84 – 1.01)	-	1.61 (1.49 – 1.75)	-	3a
wild-type MCW7259	AO	12.4 kb reporter	72	5.63 (4.83 – 6.40)	<0.0001[ad]	51.19 (47.77 – 57.25)	<0.0001 [ad]	3a
*rad51*Δ MCW9700	IO	12.4 kb reporter	24	0.03 (0.00 – 0.05)	<0.0001[f]	5.43 (4.78 – 6.38)	<0.0001[f]	3a
*rad51*Δ MCW9701	AO	12.4 kb reporter	25	1.14 (0.93 – 1.54)	<0.0001[g]	55.12 (45.76 – 62.78)	>0.9999[g]	3a
*rad51*Δ *rad52*Δ MCW7816	IO	12.4 kb reporter	16	0	<0.0001[f]	0.87 (0.43 – 1.27)	0.1551[f]	3a
*rad51*Δ *rad52*Δ MCW7818	AO	12.4 kb reporter	15	0	<0.0001[g]	0.85 (0.69 – 1.64)	<0.0001[g]	3a
*rad54*Δ MCW9030	IO	12.4 kb reporter	16	0.00 (0.00 – 0.08)	<0.0001[f]	6.86 (5.63 – 9.59)	<0.0001[f]	3a
*rad54*Δ MCW9013	AO	12.4 kb reporter	15	0.62 (0.32 – 1.42)	<0.0001[g] >0.9999 [ae]	15.51 (12.76 – 24.67)	<0.0001[g] <0.0001[ae]	3a
rad54-K300A MCW9006	IO	12.4 kb reporter	19	0.00 (0.00 – 0.09)	<0.0001[f]	3.68 (2.73 – 6.14)	0.0018[f]	3a
rad54-K300A MCW9008	AO	12.4 kb reporter	18	0.87 (0.40 – 1.92)	<0.0001[g] >0.9999[ae]	22.83 (18.95 – 33.68)	0.0001[g] 0.0004[ae]	3a
*rad55*Δ MCW7588	IO	12.4 kb reporter	30	0.00 (0.00 – 0.11)	<0.0001[f]	9.50 (7.56 – 10.76)	<0.0001[f]	3a
*rad55*Δ MCW7590	AO	12.4 kb reporter	29	1.38 (0.95 – 1.82)	<0.0001[g]	85.83 (61.89 – 94.68)	0.0039[g]	3a
*rdl1*Δ MCW8931	IO	12.4 kb reporter	15	0.67 (0.52 – 0.93)	>0.9999[f]	3.25 (2.67 – 4.05)	0.0112[f]	3a
*rdl1*Δ MCW9140	AO	12.4 kb reporter	21	2.35 (2.03 – 3.11)	0.0058[g]	48.30 (36.36 – 73.41)	>0.9999[g]	3a
wild-type *ori1253*Δ MCW8700	IO	12.4 kb reporter	44	1.76 (1.53 – 2.30)	-	3.39 (2.73 – 3.77)	-	3b, 5b
wild-type *ori1253*Δ MCW8888	AO	12.4 kb reporter	48	91.73 (75.58 – 107.8)	<0.0001[h]	733 (628.1 – 937.8)	<0.0001[h]	3b, 5b

*rad51*Δ *ori1253*Δ MCW9000	IO	12.4 kb reporter	28	0.00 (0.00 – 0.05)	<0.0001[h]<br><0.0001[j]	11.61 (10.58 – 14.34)	<0.0001[h]<br><0.0001[j]	3b, 3c, 5b
*rad51*Δ *ori1253*Δ MCW9002	AO	12.4 kb reporter	26	16.80 (10.79 – 21.21)	0.0004[h]<br><0.0001[i]<br>0.0001[k]	510.8 (416.7 – 690.0)	<0.0001[h]<br>0.0175[i]<br>0.0017[k]	3b, 3c, 5b
*rad51*Δ *rad52*Δ *ori1253*Δ MCW9210	IO	12.4 kb reporter	21	0	-	0.17 (0.14 – 0.46)	-	3b
*rad51*Δ *rad52*Δ *ori1253*Δ MCW9212	AO	12.4 kb reporter	22	0.00 (0.00 – 0.01)	<0.0001[i]	0.66 (0.39 – 1.18)	<0.0001[i]	3b
rad51+ *ori1253*Δ MCW9855	IO	12.4 kb reporter	15	1.84 (1.59 – 2.33)	-	3.44 (1.86 – 6.10)	-	3c
rad51+ *ori1253*Δ MCW9856	AO	12.4 kb reporter	19	81.08 (51.72 – 113.1)	-	924.4 (702.1 - 1780)	-	3c
rad51-3A *ori1253*Δ MCW10543	IO	12.4 kb reporter	16	0.12 (0.08– 0.22)	0.0012[j]	11.62 (10.35 – 12.57)	<0.0001[j]	3c
rad51-3A *ori1253*Δ MCW9851	AO	12.4 kb reporter	20	7.26 (3.88 – 12.35)	<0.0001[k]<br>0.0610[af]<br><0.0001[ag]	213.9 (150.9 – 277.0)	<0.0001[k]<br>0.0016[af]<br><0.0001[ag]	3c
rad52+ *ori1253*Δ MCW9679	IO	12.4 kb reporter	20	2.61 (2.12 – 3.73)	-	4.91 (4.17 – 5.86)	-	4c
rad52+ *ori1253*Δ MCW9694	AO	12.4 kb reporter	18	124.4 (79.95 – 164.4)	-	878.7 (811.0 - 1179)	-	4c
*rad52*ΔC308 *ori1253*Δ MCW9680	IO	12.4 kb reporter	21	0.16 (0.07 – 0.19)	0.0341[l]<br>0.0323[m]	9.77 (8.49 – 10.75)	0.0418[l]<br>>0.9999[m]	4c
*rad52*ΔC308 *ori1253*Δ MCW9693	AO	12.4 kb reporter	20	14.27 (11.80 – 16.78)	0.0035[n]<br>>0.9999[o]	349.3 (276.1 – 486.2)	0.0038[n]<br>0.6089[o]	4c
*rad52*ΔC208 *ori1253*Δ MCW9681	IO	12.4 kb reporter	19	0.03 (0.00 – 0.05)	<0.0001[l]<br>>0.9999[m]	1.67 (1.20 – 2.14)	0.0253[l]<br><0.0001[m]	4c
*rad52*ΔC208 *ori1253*Δ MCW9692	AO	12.4 kb reporter	19	0.05 (0.00 – 0.17)	<0.0001[n]<br>0.0034[o]	3.53 (2.34 – 5.23)	<0.0001[n]<br><0.0001[o]	4c
rad52+ *rad51*Δ *ori1253*Δ MCW9676	IO	12.4 kb reporter	20	0.00 (0.00 – 0.06)	<0.0001[l]	11.19 (9.86 – 12.02)	0.0039[l]	4c
rad52+ *rad51*Δ *ori1253*Δ MCW9691	AO	12.4 kb reporter	25	8.93 (6.94 – 12.77)	<0.0001[n]	527.0 (428.9 – 770.5)	0.4821[n]	4c
*rad52*ΔC308 *rad51*Δ *ori1253*Δ MCW9677	IO	12.4 kb reporter	21	0.00 (0.00 – 0.05)	<0.0001[l]<br>>0.9999[m]	10.30 (8.31 – 13.22)	0.0113[l]<br>>0.9999[m]	4c
*rad52*ΔC308 *rad51*Δ *ori1253*Δ MCW9690	AO	12.4 kb reporter	20	26.26 (19.42 – 43.27)	0.3495[n]<br>0.0992[o]	806.1 (675.6 - 1000)	>0.9999[n]<br>>0.9999[o]	4c
*rad52*ΔC208 *rad51*Δ	IO	12.4 kb reporter	20	0	<0.0001[l]<br>0.6130[m]	1.72 (1.45 – 2.09)	0.0381[l]<br><0.0001[m]	4c

ori1253Δ MCW9678								
*rad52Δ*C208 *rad51Δ* *ori1253Δ* MCW9689	AO	12.4 kb reporter	23	0.03 (0.02 – 0.06)	<0.0001[n] 0.0005[o]	2.45 (1.77 – 3.14)	<0.0001[n] <0.0001[o]	4c
wild-type *ori1253Δ* MCW8700 + pREP41	IO	12.4 kb reporter	25	0.81 (0.52 – 0.95)	-	3.60 (2.95 – 4.31)	-	S3c
wild-type *ori1253Δ* MCW8888 + pREP41	AO	12.4 kb reporter	26	39.38 (31.25 – 61.92)	-	837.9 (687.3 - 1216)	-	S3d
wild-type *ori1253Δ* MCW8700 + pREP41-*rad52+*	IO	12.4 kb reporter	17	0.46 (0.22 – 0.83)	0.2460[p]	4.72 (3.79 – 7.96)	0.1093[p]	S3c
wild-type *ori1253Δ* MCW8888 + pREP41-*rad52+*	AO	12.4 kb reporter	15	27.59 (20.51 – 43.90)	0.1007[q]	1109 (904.6 - 1332)	0.5283[q]	S3d
wild-type *ori1253Δ* MCW8700 + pREP41-*rad52Δ*C1-316	IO	12.4 kb reporter	19	0.54 (0.39 – 0.81)	0.3366[p]	4.10 (2.94 – 6.61)	0.7346[p]	S3c
wild-type *ori1253Δ* MCW8888 + pREP41-*rad52Δ*C1-316	AO	12.4 kb reporter	24	37.53 (28.66 – 56.67)	>0.9999[q]	1475 (1201 - 1720)	0.0005[q]	S3d
wild-type *ori1253Δ* MCW8700 + pREP41-*rad52Δ*C1-210	IO	12.4 kb reporter	23	1.10 (0.76 – 1.67)	0.1643[p]	1.33 (1.22 – 1.98)	0.0003[p]	S3c
wild-type *ori1253Δ* MCW8888 + pREP41-*rad52Δ*C1-210	AO	12.4 kb reporter	30	54.91 (41.10 – 64.71)	0.4731[q]	781.5 (511.9 – 925.4)	>0.9999[q]	S3d
rad51Δ *ori1253Δ* MCW9000 + pREP41	IO	12.4 kb reporter	24	0.01 (0.00 – 0.05)	-	10.37 (7.55 – 17.84)	-	S3c
rad51Δ *ori1253Δ* MCW9002 + pREP41	AO	12.4 kb reporter	25	2.56 (1.85 – 4.26)	-	171.7 (122.7 – 194.5)	-	S3d
rad51Δ *ori1253Δ* MCW9000 + pREP41-*rad52+*	IO	12.4 kb reporter	16	0.00 (0.00 – 0.14)	>0.9999[r]	12.40 (8.80 – 22.03)	0.6735[r]	S3c
rad51Δ *ori1253Δ* MCW9002 + pREP41-*rad52+*	AO	12.4 kb reporter	17	5.28 (3.14 – 7.29)	0.1811[s]	251.6 (193.9 – 314.0)	0.0174[s]	S3d
rad51Δ *ori1253Δ* MCW9000 +	IO	12.4 kb reporter	26	0.00 (0.00 – 0.06)	0.9246[r]	9.57 (6.72 – 15.62)	>0.9999[r]	S3c

pREP41-*rad52*ΔC1-316								
*rad51*Δ *ori1253*Δ MCW9002 + pREP41-*rad52*ΔC1-316	AO	12.4 kb reporter	19	7.14 (3.13 – 13.39)	0.0065[s]	312.5 (248.8 – 417.8)	0.0001[s]	S3d
*rad51*Δ *ori1253*Δ MCW9000 + pREP41-*rad52*ΔC1-210	IO	12.4 kb reporter	25	0.02 (0.00 – 0.03)	>0.9999[r]	7.88 (6.76 – 12.10)	0.2985[r]	S3c
*rad51*Δ *ori1253*Δ MCW9002 + pREP41-*rad52*ΔC1-210	AO	12.4 kb reporter	18	6.20 (3.23 – 10.61)	0.0196[s]	258.1 (131.9 – 368.6)	0.0549[s]	S3d
*rad51*Δ *rad52*Δ *ori1253*Δ MCW9210 + pREP41	IO	12.4 kb reporter	15	0	-	0.27 (0.12 – 0.53)	-	S3c
*rad51*Δ *rad52*Δ *ori1253*Δ MCW9212 + pREP41	AO	12.4 kb reporter	19	0.00 (0.00 – 0.03)	-	1.80 (1.35 – 3.67)	-	S3d
*rad51*Δ *rad52*Δ *ori1253*Δ MCW9210 + pREP41-*rad52+*	IO	12.4 kb reporter	16	0.00 (0.00 – 0.09)	0.0853[t]	5.86 (2.65 – 8.22)	<0.0001[t]	S3c
*rad51*Δ *rad52*Δ *ori1253*Δ MCW9212 + pREP41-*rad52+*	AO	12.4 kb reporter	18	9.60 (5.66 – 15.73)	<0.0001[u] 0.0046[ah]	304.7 (238.8 – 386.4)	<0.0001[u] 0.0014[ah]	S3d
*rad51*Δ *rad52*Δ *ori1253*Δ MCW9210 + pREP41-*rad52*ΔC1-316	IO	12.4 kb reporter	16	0.03 (0.00 – 0.04)	0.0044[t]	4.24 (3.77 – 5.64)	<0.0001[t]	S3c
*rad51*Δ *rad52*Δ *ori1253*Δ MCW9212 + pREP41-*rad52*ΔC1-316	AO	12.4 kb reporter	17	9.09 (7.65 – 13.24)	<0.0001[u] 0.0116[ah]	335.4 (303.2 – 401.7)	<0.0001[u] 0.0001[ah]	S3d
*rad51*Δ *rad52*Δ *ori1253*Δ MCW9210 + pREP41-*rad52*ΔC1-210	IO	12.4 kb reporter	20	0.00 (0.00 – 0.01)	0.7979[t]	2.45 (1.88 – 3.24)	0.0010[t]	S3c
*rad51*Δ *rad52*Δ *ori1253*Δ MCW9212 + pREP41-*rad52*ΔC1-210	AO	12.4 kb reporter	21	1.98 (0.81 – 3.21)	0.0051[u]	70.00 (57.20 – 108.2)	0.0164[u]	S3d
wild-type MCW9374	IO	12.4 kb reporter	18	1.27 (0.74 – 1.75)	-	2.55 (2.17 – 4.05)	-	5a
wild-type MCW9235	AO	12.4 kb reporter	20	9.75 (7.67 – 15.57)	-	138.4 (114.0 – 178.9)	-	5a
rad52-R45A MCW9793	IO	12.4 kb reporter	18	1.01 (0.86 – 1.18)	>0.9999[w]	3.16 (2.93 – 3.69)	0.7446[w]	5a

rad52-R45A MCW9794	AO	12.4 kb reporter	20	2.89 (2.53 – 4.02)	0.0009[x]	10.09 (7.73 – 13.04)	<0.0001[x]	5a
*rad51*Δ MCW9587	IO	12.4 kb reporter	20	0.00 (0.00 – 0.04)	<0.0001[w]	5.82 (5.10 – 6.73)	<0.0001[w]	5a
*rad51*Δ MCW9570	AO	12.4 kb reporter	20	2.97 (2.14 – 3.78)	0.0003[x]	90.17 (72.67 – 115.4)	0.3782[x]	5a
*rad51*Δ *rad52*-R45A MCW9826	IO	12.4 kb reporter	19	0	<0.0001[w]	0.76 (0.59 – 0.90)	0.0022[w]	5a
*rad51*Δ *rad52*-R45A MCW9824	AO	12.4 kb reporter	24	0	<0.0001[x]	0.96 (0.70 – 1.25)	<0.0001[x]	5a
rad52-R45A *ori1253*Δ MCW9838	IO	12.4 kb reporter	29	0.97 (0.70 – 1.09)	0.0029[h]	2.52 (1.84 – 3.10)	0.1387[h]	5b
rad52-R45A *ori1253*Δ MCW9840	AO	12.4 kb reporter	29	13.76 (11.33 – 16.80)	<0.0001[i]	22.65 (17.80 – 31.61)	<0.0001[i]	5b
*rad51*Δ *rad52*-R45A *ori1253*Δ MCW9842	IO	12.4 kb reporter	29	0	<0.0001[h]	1.96 (1.55 – 2.71)	0.0183[h]	5b
*rad51*Δ *rad52*-R45A *ori1253*Δ MCW9844	AO	12.4 kb reporter	25	0.00 (0.00 – 0.02)	<0.0001[i]	2.86 (2.33 – 3.60)	<0.0001[i]	5b
wild-type MCW7132	IO	0.2 kb reporter	29	1.06 (0.81 – 1.40)	-	4.43 (3.71 – 5.29)	-	5c
wild-type MCW7134	AO	0.2 kb reporter	39	101.8 (87.38 – 119.5)	-	652.6 (501.3 – 723.2)	-	5c
rad52-R45A MCW9715	IO	0.2 kb reporter	20	0.94 (0.81 – 1.47)	>0.9999[y]	5.42 (4.58 – 7.21)	0.1636[y]	5c
rad52-R45A MCW9718	AO	0.2 kb reporter	20	51.10 (39.01 – 56.63)	0.0025[z]	119.4 (101.7 – 125.5)	<0.0001[z]	5c
*rad51*Δ MCW8788	IO	0.2 kb reporter	18	0.01 (0.00 – 0.04)	<0.0001[y]	11.50 (8.91 – 12.58)	<0.0001[y]	5c
*rad51*Δ MCW8790	AO	0.2 kb reporter	18	18.53 (13.08 – 19.61)	<0.0001[z]	326.6 (260.7 – 415.4)	0.0062[z]	5c
*rad51*Δ *rad52*-R45A MCW9714	IO	0.2 kb reporter	20	0.00 (0.00 – 0.01)	<0.0001[y]	2.45 (1.90 – 3.24)	0.0103[y]	5c
*rad51*Δ *rad52*-R45A MCW9717	AO	0.2 kb reporter	20	0.02 (0.00 – 0.03)	<0.0001[z]	3.73 (2.94 – 4.94)	<0.0001[z]	5c

[a] The different recombination reporters are shown in Figure 1.
[b] The values in parentheses are the 95% confidence interval.
[c] Approximate p-values are calculated by the Kruskal-Wallis test (one-way ANOVA on ranks) with Dunn's multiple comparisons post-test unless otherwise stated.
[d] Versus the equivalent recombinant frequency of MCW4712.
[e] Versus the equivalent recombinant frequency of MCW4713.
[f] Versus the equivalent recombinant frequency of MCW7257.
[g] Versus the equivalent recombinant frequency of MCW7259.
[h] Versus the equivalent recombinant frequency of MCW8700.
[i] Versus the equivalent recombinant frequency of MCW8888.

[j] Versus the equivalent recombinant frequency of MCW9855.
[k] Versus the equivalent recombinant frequency of MCW9856.
[l] Versus the equivalent recombinant frequency of MCW9679.
[m] Versus the equivalent recombinant frequency of MCW9676.
[n] Versus the equivalent recombinant frequency of MCW9694.
[o] Versus the equivalent recombinant frequency of MCW9691.
[p] Versus the equivalent recombinant frequency of MCW8700 + pREP41.
[q] Versus the equivalent recombinant frequency of MCW8888 + pREP41.
[r] Versus the equivalent recombinant frequency of MCW9000 + pREP41.
[s] Versus the equivalent recombinant frequency of MCW9002 + pREP41.
[t] Versus the equivalent recombinant frequency of MCW9210 + pREP41.
[u] Versus the equivalent recombinant frequency of MCW9212 + pREP41.
[w] Versus the equivalent recombinant frequency of MCW9374.
[x] Versus the equivalent recombinant frequency of MCW9235.
[y] Versus the equivalent recombinant frequency of MCW7132.
[z] Versus the equivalent recombinant frequency of MCW7134.
[aa] Versus the equivalent recombinant frequency of MCW1692.
[ab] Versus the equivalent recombinant frequency of MCW9928 – exact p-value calculated by the two-tailed Mann Whitney test.
[ac] Versus the equivalent recombinant frequency of MCW9931 – exact p-value calculated by the two-tailed Mann Whitney test.
[ad] Versus the equivalent recombinant frequency of MCW7257 – exact p-value calculated by the two-tailed Mann Whitney test.
[ae] Versus the equivalent recombinant frequency of MCW9701.
[af] Versus the equivalent recombinant frequency of MCW9002.
[ag] Versus the equivalent recombinant frequency of MCW9855 – exact p-value calculated by the two-tailed Mann Whitney test.
[ah] Versus the equivalent recombinant frequency of MCW9212 + pREP41ΔC1-210

Supplementary Table 2: Recombination frequencies

Relevant Genotype and Strain no.	*RTS1* presence and orientation	Recomb-ination reporter[a]	Number of colonies analysed (*n*)	$Ura^- His^+$ recombinant frequency (x 10^{-4})		$Ura^- His^-$ recombinant frequency (x 10^{-4})		Figure
				Median (95% CI)[b]	p-value[c]	Median (95% CI)[b]	p-value[c]	
wild-type MCW9276	IO	12.4 kb *Alu*Sx1-*Alu*Sp reporter	99	0.000 (0.000 – 0.000)	-	0.000 (0.000 – 0.000)	-	6b
wild-type MCW9278	AO	12.4 kb *Alu*Sx1-*Alu*Sp reporter	96	0.024 (0.015 – 0.044)	<0.0001[d]	0.101 (0.074 – 0.130)	<0.0001[d]	6b
rad52-R45A MCW10086	IO	12.4 kb *Alu*Sx1-*Alu*Sp reporter	24	0.000 (0.000 – 0.000)	0.5482[d]	0.000 (0.000 – 0.000)	>0.9999[d]	6b
rad52-R45A (isolate 1) MCW10083	AO	12.4 kb *Alu*Sx1-*Alu*Sp reporter	24	0.000 (0.000 – 0.004)	>0.9999[d] 0.0004[e]	0.000 (0.000 – 0.000)	>0.9999[d] <0.0001[e]	6b
rad52-R45A (isolate 2) MCW10084	AO	12.4 kb *Alu*Sx1-*Alu*Sp reporter	23	0.000 (0.000 – 0.015)	>0.9999[d] 0.0011[e]	0.000 (0.000 – 0.004)	>0.9999[d] <0.0001[e]	6b

[a] The recombination reporter is shown in Figure 6A.
[b] The values in parentheses are the 95% confidence interval.
[c] Approximate p-values are calculated by the Kruskal-Wallis test (one-way ANOVA on ranks) with Dunn's multiple comparisons post-test.
[d] Versus the equivalent recombinant frequency of MCW9276.
[e] Versus the equivalent recombinant frequency of MCW9278.

Supplementary Table 3: ***Schizosaccharomyces pombe*** **strains** (in order of appearance)

Strain No.	Relevant genotype	Source
MCW4712	*h⁺ ade6-M375* int::*pUC8*/*his3+*/*RTS1*-IO/*ade6-L469 ura4-D18 leu1-32 his3-D1 arg3-D4*	(Ahn et al., 2005)
MCW4713	*h⁺ ade6-M375* int::*pUC8*/*his3+*/*RTS1*-AO/*ade6-L469 ura4-D18 leu1-32 his3-D1 arg3-D4*	(Ahn *et al.*, 2005)
MCW1691	*h⁺ rad51Δ::arg3+ ade6-M375* int::*pUC8*/*his3+*/*RTS1*-IO/*ade6-L469 ura4-D18 leu1-32 his3-D1 arg3-D4*	(Ahn *et al.*, 2005)
MCW1692	*h⁺ rad51Δ::arg3+ ade6-M375* int::*pUC8*/*his3+*/*RTS1*-AO/*ade6-L469 ura4-D18 leu1-32 his3-D1 arg3-D4*	(Ahn *et al.*, 2005)
MCW1695	*h⁺ rad51Δ::arg3+ rad52Δ::ura4+ ade6-M375* int::*pUC8*/*his3+*/*RTS1*-IO/*ade6-L469 ura4-D18 leu1-32 his3-D1 arg3-D4*	(Ahn *et al.*, 2005)
MCW1696	*h⁺ rad51Δ::arg3+ rad52Δ::ura4+ ade6-M375* int::*pUC8*/*his3+*/*RTS1*-AO/*ade6-L469 ura4-D18 leu1-32 his3-D1 arg3-D4*	(Ahn *et al.*, 2005)
MCW9607	*h⁻ rad52Δ::rad52*-R45A-*kanMX6 ade6-M375* int::pUC8/*his3+*/*RTS1*-IO/*ade6-L469 ura4-D18 leu1-32 his3-D1 arg3-D4*	This study
MCW9608	*h⁻ rad52Δ::rad52*-R45A-*kanMX6 ade6-M375* int::pUC8/*his3+*/*RTS1*-AO/*ade6-L469 ura4-D18 leu1-32 his3-D1 arg3-D4*	This study
MCW9723	*h⁻ rad51Δ::arg3+ rad52Δ::rad52*-R45A-*kanMX6 ade6-M375* int::pUC8/*his3+*/*RTS1*-IO/*ade6-L469 ura4-D18 leu1-32 his3-D1 arg3-D4*	This study
MCW9725	*h⁻ rad51Δ::arg3+ rad52Δ::rad52*-R45A-*kanMX6 ade6-M375* int::pUC8/*his3+*/*RTS1*-AO/*ade6-L469 ura4-D18 leu1-32 his3-D1 arg3-D4*	This study
MCW2651	*h⁺ rad54Δ::ura4+ ade6-M375* int::*pUC8*/*his3+*/*RTS1*-IO/*ade6-L469 ura4-D18 leu1-32 his3-D1 arg3-D4*	This study
MCW8392	*h⁺ rad54Δ::kanMX6 ade6-M375* int::pUC8/*his3+*/*RTS1*-AO/*ade6-L469 ura4-D18 leu1-32 his3-D1 arg3-D4*	This study
MCW7034	*h⁺ rad54Δ::rad54K300A-natMX4 ade6-M375* int::*pUC8*/*his3+*/*RTS1*-IO/*ade6-L469 ura4-D18 leu1-32 his3-D1 arg3-D4*	This study
MCW7037	*h⁺ rad54Δ::rad54K300A-natMX4 ade6-M375* int::*pUC8*/*his3+*/*RTS1*-AO/*ade6-L469 ura4-D18 leu1-32 his3-D1 arg3-D4*	This study
MCW2650	*h⁺ rad55Δ::arg3+ ade6-M375* int::*pUC8*/*his3+*/*RTS1*-IO/*ade6-L469 ura4-D18 leu1-32 his3-D1 arg3-D4*	This study
MCW2655	*h⁺ rad55Δ::arg3+ ade6-M375* int::*pUC8*/*his3+*/*RTS1*-AO/*ade6-L469 ura4-D18 leu1-32 his3-D1 arg3-D4*	This study
MCW6539	*h⁺ rad57Δ::LEU2 ade6-M375* int::*pUC8*/*his3+*/*RTS1*-IO/*ade6-L469 ura4-D18 leu1-32 his3-D1 arg3-D4*	This study
MCW6454	*h⁺ rad57Δ::LEU2 ade6-M375* int::*pUC8*/*his3+*/*RTS1*-AO/*ade6-L469 ura4-D18 leu1-32 his3-D1 arg3-D4*	This study
MCW2647	*h⁺ swi5Δ::ura4+ ade6-M375* int::*pUC8*/*his3+*/*RTS1*-IO/*ade6-L469 ura4-D18 leu1-32 his3-D1 arg3-D4*	This study
MCW2653	*h⁺ swi5Δ::ura4+ ade6-M375* int::*pUC8*/*his3+*/*RTS1*-AO/*ade6-L469 ura4-D18 leu1-32 his3-D1 arg3-D4*	This study
MCW6497	*h⁺ sfr1Δ::hphMX4 ade6-M375* int::*pUC8*/*his3+*/*RTS1*-IO/*ade6-L469 ura4-D18 leu1-32 his3-D1 arg3-D4*	This study
MCW6499	*h⁺ sfr1Δ::hphMX4 ade6-M375* int::*pUC8*/*his3+*/*RTS1*-AO/*ade6-L469 ura4-D18 leu1-32 his3-D1 arg3-D4*	This study
MCW6501	*h⁺ rdl1Δ::natMX6 ade6-M375* int::*pUC8*/*his3+*/*RTS1*-IO/*ade6-L469 ura4-D18 leu1-32 his3-D1 arg3-D4*	This study
MCW6468	*h⁺ rdl1Δ::natMX6 ade6-M375* int::*pUC8*/*his3+*/*RTS1*-AO/*ade6-L469 ura4-D18 leu1-32 his3-D1 arg3-D4*	This study
MCW6505	*h⁺ rlp1Δ::natMX6 ade6-M375* int::*pUC8*/*his3+*/*RTS1*-IO/*ade6-L469 ura4-D18 leu1-32 his3-D1 arg3-D4*	This study
MCW6507	*h⁺ rlp1Δ::natMX6 ade6-M375* int::*pUC8*/*his3+*/*RTS1*-AO/*ade6-L469 ura4-D18 leu1-32 his3-D1 arg3-D4*	This study

MCW6503	*h*+ *sws1Δ*::*kanMX6 ade6-M375* int::pUC8/*his3+*/*RTS1*-IO/*ade6-L469 ura4-D18 leu1-32 his3-D1 arg3-D4*	This study
MCW6470	*h*+ *sws1Δ*::*kanMX6 ade6-M375* int::pUC8/*his3+*/*RTS1*-AO/*ade6-L469 ura4-D18 leu1-32 his3-D1 arg3-D4*	This study
MCW2912	*h*+ *rad55Δ*::*arg3+ swi5Δ*::*ura4+ ade6-M375* int::*pUC8*/*his3+*/*RTS1*-IO/*ade6-L469 ura4-D18 leu1-32 his3-D1 arg3-D4*	This study
MCW2916	*h*+ *rad55Δ*::*arg3+ swi5Δ*::*ura4+ ade6-M375* int::*pUC8*/*his3+*/*RTS1*-AO/*ade6-L469 ura4-D18 leu1-32 his3-D1 arg3-D4*	This study
MCW6828	*h*+ *rad55Δ*::*hphMX4 rdl1Δ::natMX6 ade6-M375* int::*pUC8*/*his3+*/*RTS1*-IO/*ade6-L469 ura4-D18 leu1-32 his3-D1 arg3-D4*	This study
MCW6832	*h*+ *rad55Δ*::*hphMX4 rdl1Δ::natMX6 ade6-M375* int::*pUC8*/*his3+*/*RTS1*-AO/*ade6-L469 ura4-D18 leu1-32 his3-D1 arg3-D4*	This study
MCW7240	*h*+ *rdl1Δ*::*natMX6 swi5Δ*::*ura4+ ade6-M375* int::*pUC8*/*his3+*/*RTS1*-IO/*ade6-L469 ura4-D18 leu1-32 his3-D1 arg3-D4*	This study
MCW7242	*h*+ *rdl1Δ*::*natMX6 swi5Δ*::*ura4+ ade6-M375* int::*pUC8*/*his3+*/*RTS1*-AO/*ade6-L469 ura4-D18 leu1-32 his3-D1 arg3-D4*	This study
MCW7250	*h*+ *rad55Δ*::*arg3+ rdl1Δ*::*natMX6 swi5Δ*::*ura4+ ade6-M375* int::*pUC8*/*his3+*/*RTS1*-IO/*ade6-L469 ura4-D18 leu1-32 his3-D1 arg3-D4*	This study
MCW7252	*h*+ *rad55Δ*::*arg3+ rdl1Δ*::*natMX6 swi5Δ*::*ura4+ ade6-M375* int::*pUC8*/*his3+*/*RTS1*-AO/*ade6-L469 ura4-D18 leu1-32 his3-D1 arg3-D4*	This study
MCW6430	*h*+ *rad51Δ*::*arg3+ rad55Δ*::*hphMX4 ade6-M375* int::*pUC8*/*his3+*/*RTS1*-IO/*ade6-L469 ura4-D18 leu1-32 his3-D1 arg3-D4*	This study
MCW6565	*h*+ *rad51Δ*::*arg3+ rad55Δ*::*hphMX4 ade6-M375* int::*pUC8*/*his3+*/*RTS1*-AO/*ade6-L469 ura4-D18 leu1-32 his3-D1 arg3-D4*	This study
MCW6836	*h*+ *rad51Δ*::*arg3+ swi5Δ::ura4+ ade6-M375* int::*pUC8*/*his3+*/*RTS1*-IO/*ade6-L469 ura4-D18 leu1-32 his3-D1 arg3-D4*	This study
MCW6842	*h*+ *rad51Δ*::*arg3+ swi5Δ::ura4+ ade6-M375* int::*pUC8*/*his3+*/*RTS1*-AO/*ade6-L469 ura4-D18 leu1-32 his3-D1 arg3-D4*	This study
MCW9928	*h*? *rad51Δ*::*rad51+-kanMX6 ade6-M375* int::*pUC8*/*his3+*/*RTS1*-IO/*ade6-L469 ura4-D18 leu1-32 his3-D1 arg3-D4*	This study
MCW9931	*h*? *rad51Δ*::*rad51+-kanMX6 ade6-M375* int::*pUC8*/*his3+*/*RTS1*-AO/*ade6-L469 ura4-D18 leu1-32 his3-D1 arg3-D4*	This study
MCW9904	*h*? *rad51Δ*::*rad51*-R152A-R324A-K334A-*kanMX6 ade6-M375* int::*pUC8*/*his3+*/*RTS1*-IO/*ade6-L469 ura4-D18 leu1-32 his3-D1 arg3-D4*	This study
MCW9906	*h*? *rad51Δ*::*rad51*-R152A-R324A-K334A-*kanMX6 ade6-M375* int::*pUC8*/*his3+*/*RTS1*-AO/*ade6-L469 ura4-D18 leu1-32 his3-D1 arg3-D4*	This study
MCW1221	*h*+ *ura4-D18 leu1-32 his3-D1 arg3-D4*	Lab strain
MCW6910	*h*+ *rad54Δ*::*rad54+-natMX6 ura4-D18 leu1-32 his3-D1 arg3-D4*	This study
MCW1230	*h*+ *rad54Δ*::*ura4+ ura4-D18 leu1-32 his3-D1 arg3-D4*	Lab strain
MCW6911	*h*+ *rad54Δ*::*rad54*-K300A-*natMX6 ura4-D18 leu1-32 his3-D1 arg3-D4*	This study
MCW7257	*h*+ *ade6Δ*::*RTS1*-IO-*hphMX4* (12.4 kb from *RTS1*-IO-*hphMX4*) int::*ade6-L469*/pUC8/*his3+*/*ade6-M375*/*kanMX6 ura4-D18 leu1-32 his3-D1 arg3-D4*	(Nguyen et al., 2015)
MCW7259	*h*+ *ade6Δ*::*RTS1*-AO-*hphMX4* (12.4 kb from *RTS1*-AO-*hphMX4*) int::*ade6-L469*/pUC8/*his3+*/*ade6-M375*/*kanMX6 ura4-D18 leu1-32 his3-D1 arg3-D4*	(Nguyen *et al.*, 2015)
MCW9700	*h*- *rad51Δ*::*arg3+ ade6Δ::RTS1*-IO-*hphMX4* (12.4 kb from *RTS1*-IO-*hphMX4*) *int::ade6-L469*/pUC8/*his3+*/*ade6-M375*/*kanMX6 ura4-D18 leu1-32 his3-D1 arg3-D4*	This study
MCW9701	*h*+ *rad51Δ*::*arg3+ ade6Δ::RTS1*-AO-*hphMX4* (12.4 kb from *RTS1*-AO-*hphMX4*) *int::ade6-L469*/pUC8/*his3+*/*ade6-M375*/*kanMX6 ura4-D18 leu1-32 his3-D1 arg3-D4*	This study
MCW7816	*h*+ *rad51Δ*::*arg3+ rad52Δ*::*ura4+ ade6Δ*::*RTS1*-IO-*hphMX4* (12.4 kb from *RTS1*-IO-*hphMX4*) int::*ade6-L469*/pUC8/*his3+*/*ade6-M375*/*kanMX6 ura4-D18 leu1-32 his3-D1 arg3-D4*	This study

MCW7818	*h*$^{+}$ *rad51*Δ::*arg3*+ *rad52*Δ::*ura4*+ *ade6*Δ::*RTS1*-AO-*hphMX4* (12.4 kb from *ade6*) int::*ade6-L469*/pUC8/*his3+*/*ade6-M375*/*kanMX6 ura4-D18 leu1-32 his3-D1 arg3-D4*	This study
MCW9030	*h*$^{+}$ *rad54*Δ::*kanMX6 ade6*Δ::*RTS1*-IO-*hphMX4* (12.4 kb from *ade6*) int::*ade6-L469*/pUC8/*his3+*/*ade6-M375*/*kanMX6 ura4-D18 leu1-32 his3-D1 arg3-D4*	This study
MCW9013	*h*$^{-}$ *rad54*Δ::*kanMX6 ade6*Δ::*RTS1*-AO-*hphMX4* (12.4 kb from *ade6*) int::*ade6-L469*/pUC8/*his3+*/*ade6-M375*/*kanMX6 ura4-D18 leu1-32 his3-D1 arg3-D4*	This study
MCW9006	*h*$^{+}$ *rad54*Δ::*rad54*-K300A-*natMX4 ade6*Δ::*RTS1*-IO-*hphMX4* (12.4 kb from *ade6*) int::*ade6-L469*/pUC8/*his3+*/*ade6-M375*/*kanMX6 ura4-D18 leu1-32 his3-D1 arg3-D4*	This study
MCW9008	*h*$^{-}$ *rad54*Δ::*rad54*-K300A-*natMX4 ade6*Δ::*RTS1*-AO-*hphMX4* (12.4 kb from *ade6*) int::*ade6-L469*/pUC8/*his3+*/*ade6-M375*/*kanMX6 ura4-D18 leu1-32 his3-D1 arg3-D4*	This study
MCW7588	*h*$^{+}$ *rad55*Δ::*arg3*+ *ade6*Δ::*RTS1*-IO-*hphMX4* (12.4 kb from *ade6*) int::*ade6-L469*/pUC8/*his3+*/*ade6-M375*/*kanMX6 ura4-D18 leu1-32 his3-D1 arg3-D4*	This study
MCW7590	*h*$^{+}$ *rad55*Δ::*arg3*+ *ade6*Δ::*RTS1*-AO-*hphMX4* (12.4 kb from *ade6*) int::*ade6-L469*/pUC8/*his3+*/*ade6-M375*/*kanMX6 ura4-D18 leu1-32 his3-D1 arg3-D4*	This study
MCW8931	*h*$^{+}$ *rdl1*Δ::*natMX6 ade6*Δ::*RTS1*-IO-*hphMX4* (12.4 kb from *ade6*) int::*ade6-L469*/pUC8/*his3+*/*ade6-M375*/*kanMX6 ura4-D18 leu1-32 his3-D1 arg3-D4*	This study
MCW9140	*h*$^{+}$ *rdl1*Δ::*natMX6 ade6*Δ::*RTS1*-AO-*hphMX4* (12.4 kb from *ade6*) int::*ade6-L469*/pUC8/*his3+*/*ade6-M375*/*kanMX6 ura4-D18 leu1-32 his3-D1 arg3-D4*	This study
MCW8700	*h*$^{+}$ *oriIII-1253*Δ::*natMX4 ade6*Δ::*RTS1*-IO-*hphMX4* (12.4 kb from *ade6*) int::*ade6-L469/pUC8/his3+/ade6-M375/ura4+ ura4-D18 leu1-32 his3-D1 arg3-D4*	This study
MCW8888	*h*$^{+}$ *oriIII-1253*Δ::*natMX4 ade6*Δ::*RTS1*-AO-*hphMX4* (12.4 kb from *ade6*) int::*ade6-L469/pUC8/his3+/ade6-M375/ura4+ ura4-D18 leu1-32 his3-D1 arg3-D4*	This study
MCW9000	*h*$^{+}$ *rad51*Δ::*arg3*+ *oriIII-1253*Δ::*natMX4 ade6*Δ::*RTS1*-IO-*hphMX4* (12.4 kb from *ade6*) int::*ade6-L469/pUC8/his3+/ade6-M375/ura4+ ura4-D18 leu1-32 his3-D1 arg3-D4*	This study
MCW9002	*h*$^{+}$ *rad51*Δ::*arg3*+ *oriIII-1253*Δ::*natMX4 ade6*Δ::*RTS1*-AO-*hphMX4* (12.4 kb from *ade6*) int::*ade6-L469/pUC8/his3+/ade6-M375/ura4+ ura4-D18 leu1-32 his3-D1 arg3-D4*	This study
MCW9210	*h*$^{+}$ *rad51*Δ::*arg3*+ *rad52*Δ::*kanMX6 oriIII-1253*Δ::*natMX4 ade6*Δ::*RTS1*-IO-*hphMX4* (12.4 kb from *ade6*) int::*ade6-L469*/pUC8/*his3+*/*ade6-M375*/*ura4+ ura4-D18 leu1-32 his3-D1 arg3-D4*	This study
MCW9212	*h*$^{+}$ *rad51*Δ::*arg3*+ *rad52*Δ::*kanMX6 oriIII-1253*Δ::*natMX4 ade6*Δ::*RTS1*-AO-*hphMX4* (12.4 kb from *ade6*) int::*ade6-L469*/pUC8/*his3+*/*ade6-M375*/*ura4+ ura4-D18 leu1-32 his3-D1 arg3-D4*	This study
MCW9855	*h*$^{?}$ *rad51*Δ::*rad51+-kanMX6 oriIII-1253*Δ::*natMX4 ade6*Δ::*RTS1*-IO-*hphMX4* (12.4 kb from *ade6*) int::*ade6-L469*/pUC8/*his3+*/*ade6-M375*/*ura4+ ura4-D18 leu1-32 his3-D1 arg3-D4*	This study
MCW9856	*h*$^{?}$ *rad51*Δ::*rad51+-kanMX6 oriIII-1253*Δ::*natMX4 ade6*Δ::*RTS1*-AO-*hphMX4* (12.4 kb from *ade6*) int::*ade6-L469*/pUC8/*his3+*/*ade6-M375*/*ura4+ ura4-D18 leu1-32 his3-D1 arg3-D4*	This study
MCW10543	*h*$^{+}$ *rad51*Δ::*rad51*-R152A-R324A-K334A-*kanMX6 oriIII-1253*Δ::*natMX4 ade6*Δ::*RTS1*-IO-*hphMX4* (12.4 kb from *ade6*) int::*ade6-L469*/pUC8/*his3+*/*ade6-M375*/*ura4+ ura4-D18 leu1-32 his3-D1 arg3-D4*	This study
MCW9851	*h*$^{?}$ *rad51*Δ::*rad51*-R152A-R324A-K334A-*kanMX6 oriIII-1253*Δ::*natMX4 ade6*Δ::*RTS1*-AO-*hphMX4* (12.4 kb from *ade6*) int::*ade6-L469*/pUC8/*his3+*/*ade6-M375*/*ura4+ ura4-D18 leu1-32 his3-D1 arg3-D4*	This study
MCW9679	*h*$^{?}$ *rad52*Δ::*rad52+-kanMX6 oriIII-1253*Δ::*natMX4 ade6*Δ::*RTS1*-IO-*hphMX4* (12.4 kb from *ade6*) int::*ade6-L469*/pUC8*/his3+/ade6-M375/ura4+ ura4-D18 leu1-32 his3-D1 arg3-D4*	This study
MCW9694	*h*$^{?}$ *rad52*Δ::*rad52+-kanMX6 oriIII-1253*Δ::*natMX4 ade6*Δ::*RTS1*-AO-*hphMX4* (12.4 kb from *ade6*) int::*ade6-L469*/pUC8/*his3+*/*ade6-M375*/*ura4+ ura4-D18 leu1-32 his3-D1 arg3-D4*	This study

MCW9680	$h^{?}$ rad52Δ::rad52ΔC1-308-kanMX6 oriIII-1253Δ::natMX4 ade6Δ::RTS1-IO-hphMX4 (12.4 kb from ade6) int::ade6-L469/pUC8/his3+/ade6-M375/ura4+ ura4-D18 leu1-32 his3-D1 arg3-D4	This study
MCW9693	$h^{?}$ rad52Δ::rad52ΔC1-308-kanMX6 oriIII-1253Δ::natMX4 ade6Δ::RTS1-AO-hphMX4 (12.4 kb from ade6) int::ade6-L469/pUC8/his3+/ade6-M375/ura4+ ura4-D18 leu1-32 his3-D1 arg3-D4	This study
MCW9681	$h^{?}$ rad52Δ::rad52ΔC1-208-kanMX6 oriIII-1253Δ::natMX4 ade6Δ::RTS1-IO-hphMX4 (12.4 kb from ade6) int::ade6-L469/pUC8/his3+/ade6-M375/ura4+ ura4-D18 leu1-32 his3-D1 arg3-D4	This study
MCW1285	h^{+} rad52Δ::ura4+ ura4-D18 leu1-32 his3-D1 arg3-D4	(Doe et al., 2004)
MCW9692	$h^{?}$ rad52Δ::rad52ΔC1-208-kanMX6 oriIII-1253Δ::natMX4 ade6Δ::RTS1-AO-hphMX4 (12.4 kb from ade6) int::ade6-L469/pUC8/his3+/ade6-M375/ura4+ ura4-D18 leu1-32 his3-D1 arg3-D4	This study
MCW9676	$h^{?}$ rad51Δ::arg3+ rad52Δ::rad52+-kanMX6 oriIII-1253Δ::natMX4 ade6Δ::RTS1-IO-hphMX4 (12.4 kb from ade6) int::ade6-L469/pUC8/his3+/ade6-M375/ura4+ ura4-D18 leu1-32 his3-D1 arg3-D4	This study
MCW9691	$h^{?}$ rad51Δ::arg3+ rad52Δ::rad52+-kanMX6 oriIII-1253Δ::natMX4 ade6Δ::RTS1-AO-hphMX4 (12.4 kb from ade6) int::ade6-L469/pUC8/his3+/ade6-M375/ura4+ ura4-D18 leu1-32 his3-D1 arg3-D4	This study
MCW9677	$h^{?}$ rad51Δ::arg3+ rad52Δ::rad52ΔC1-308-kanMX6 oriIII-1253Δ::natMX4 ade6Δ::RTS1-IO-hphMX4 (12.4 kb from ade6) int::ade6-L469/pUC8/his3+/ade6-M375/ura4+ ura4-D18 leu1-32 his3-D1 arg3-D4	This study
MCW9690	$h^{?}$ rad51Δ::arg3+ rad52Δ::rad52ΔC1-308-kanMX6 oriIII-1253Δ::natMX4 ade6Δ::RTS1-AO-hphMX4 (12.4 kb from ade6) int::ade6-L469/pUC8/his3+/ade6-M375/ura4+ ura4-D18 leu1-32 his3-D1 arg3-D4	This study
MCW9678	$h^{?}$ rad51Δ::arg3+ rad52Δ::rad52ΔC1-208-kanMX6 oriIII-1253Δ::natMX4 ade6Δ::RTS1-IO-hphMX4 (12.4 kb from ade6) int::ade6-L469/pUC8/his3+/ade6-M375/ura4+ ura4-D18 leu1-32 his3-D1 arg3-D4	This study
MCW9689	$h^{?}$ rad51Δ::arg3+ rad52Δ::rad52ΔC1-208-kanMX6 oriIII-1253Δ::natMX4 ade6Δ::RTS1-AO-hphMX4 (12.4 kb from ade6) int::ade6-L469/pUC8/his3+/ade6-M375/ura4+ ura4-D18 leu1-32 his3-D1 arg3-D4	This study
MCW9374	h^{+} ade6Δ::RTS1-IO-hphMX4 (12.4 kb from ade6) int::ade6-L469/pUC8/his3+/ade6-M375/ura4+ ura4-D18 leu1-32 his3-D1 arg3-D4	This study
MCW9235	h^{+} ade6Δ::RTS1-AO-hphMX4 (12.4 kb from ade6) int::ade6-L469/pUC8/his3+/ade6-M375/ura4+ ura4-D18 leu1-32 his3-D1 arg3-D4	This study
MCW9793	h^{+} rad52Δ::rad52-R45A-kanMX6 ade6Δ::RTS1-IO-hphMX4 (12.4 kb from ade6) int::ade6-L469/pUC8/his3+/ade6-M375/ura4+ ura4-D18 leu1-32 his3-D1 arg3-D4	This study
MCW9794	h^{+} rad52Δ::rad52-R45A-kanMX6 ade6Δ::RTS1-AO-hphMX4 (12.4 kb from ade6) int::ade6-L469/pUC8/his3+/ade6-M375/ura4+ ura4-D18 leu1-32 his3-D1 arg3-D4	This study
MCW9587	h^{+} rad51Δ::arg3+ ade6Δ::RTS1-IO-hphMX4 (12.4 kb from ade6) int::ade6-L469/pUC8/his3+/ade6-M375/ura4+ ura4-D18 leu1-32 his3-D1 arg3-D4	This study
MCW9570	h^{-} rad51Δ::arg3+ ade6Δ::RTS1-AO-hphMX4 (12.4 kb from ade6) int::ade6-L469/pUC8/his3+/ade6-M375/ura4+ ura4-D18 leu1-32 his3-D1 arg3-D4	This study
MCW9826	$h^{?}$ rad51Δ::arg3+ rad52Δ::rad52-R45A-kanMX6 ade6Δ::RTS1-IO-hphMX4 (12.4 kb from ade6) int::ade6-L469/pUC8/his3+/ade6-M375/ura4+ ura4-D18 leu1-32 his3-D1 arg3-D4	This study
MCW9824	h^{+} rad51Δ::arg3+ rad52Δ::rad52-R45A-kanMX6 ade6Δ::RTS1-AO-hphMX4 (12.4 kb from ade6) int::ade6-L469/pUC8/his3+/ade6-M375/ura4+ ura4-D18 leu1-32 his3-D1 arg3-D4	This study
MCW9838	$h^{?}$ rad52Δ::rad52-R45A-kanMX6 oriIII-1253Δ::natMX4 ade6Δ::RTS1-IO-hphMX4 (12.4 kb from ade6) int::ade6-L469/pUC8/his3+/ade6-M375/ura4+ ura4-D18 leu1-32 his3-D1 arg3-D4	This study
MCW9840	$h^{?}$ rad52Δ::rad52-R45A-kanMX6 oriIII-1253Δ::natMX4 ade6Δ::RTS1-AO-hphMX4 (12.4 kb from ade6) int::ade6-L469/pUC8/his3+/ade6-M375/ura4+ ura4-D18 leu1-32 his3-D1 arg3-D4	This study

MCW9842	*h? rad51Δ::arg3+ rad52Δ::rad52*-R45A-*kanMX6 oriIII-1253Δ::natMX4 ade6Δ::RTS1*-IO-*hphMX4* (12.4 kb from *ade6*) int::*ade6-L469*/pUC8/*his3+*/*ade6-M375*/*ura4+ ura4-D18 leu1-32 his3-D1 arg3-D4*	This study
MCW9844	*h? rad51Δ::arg3+ rad52Δ::rad52*-R45A-*kanMX6 oriIII-1253Δ::natMX4 ade6Δ::RTS1*-AO-*hphMX4* (12.4 kb from *ade6*) int::*ade6-L469*/pUC8/*his3+*/*ade6-M375*/*ura4+ ura4-D18 leu1-32 his3-D1 arg3-D4*	This study
MCW7132	*h⁻ ade6-M375* int::pUC8/*his3+*/*ade6-L469* (0.2 kb from *ade6-M375*) int::*RTS1*-IO-*hphMX4 ura4-D18 leu1-32 his3-D1 arg3-D4*	(Jalan et al., 2019)
MCW7134	*h⁻ ade6-M375* int::*pUC8*/*his3+*/*ade6-L469* (0.2 kb from *ade6-M375*) int::*RTS1*-AO-*hphMX4 ura4-D18 leu1-32 his3-D1 arg3-D4*	(Jalan *et al.*, 2019)
MCW9715	*h⁻ rad52Δ::rad52*-R45A-*kanMX6 ade6-M375* int::pUC8/*his3+*/*ade6-L469* (0.2 kb from *ade6-M375*) int::*RTS1*-IO-*hphMX4 ura4-D18 leu1-32 his3-D1 arg3-D4*	This study
MCW9718	*h⁺ rad52Δ::rad52*-R45A-*kanMX6 ade6-M375* int::pUC8/*his3+*/*ade6-L469* (0.2 kb from *ade6-M375*) int::*RTS1*-AO-*hphMX4 ura4-D18 leu1-32 his3-D1 arg3-D4*	This study
MCW8788	*h⁺ rad51Δ::arg3+ ade6-M375* int::pUC8/*his3+*/*ade6-L469* (0.2 kb from *ade6-M375*) int::*RTS1*-IO-*hphMX4 ura4-D18 leu1-32 his3-D1 arg3-D4*	This study
MCW8790	*h⁺ rad51Δ::arg3+ ade6-M375* int::*pUC8*/*his3+*/*ade6-L469* (0.2 kb from *ade6-M375*) int::*RTS1*-AO-*hphMX4 ura4-D18 leu1-32 his3-D1 arg3-D4*	This study
MCW9714	*h⁻ rad51Δ::arg3+ rad52Δ::rad52*-R45A-*kanMX6 ade6-M375* int::pUC8/*his3+*/*ade6-L469* (0.2 kb from *ade6-M375*) int::*RTS1*-IO-*hphMX4 ura4-D18 leu1-32 his3-D1 arg3-D4*	This study
MCW9717	*h⁻ rad51Δ::arg3+ rad52Δ::rad52*-R45A-*kanMX6 ade6-M375* int::pUC8/*his3+*/*ade6-L469* (0.2 kb from *ade6-M375*) int::*RTS1*-AO-*hphMX4 ura4-D18 leu1-32 his3-D1 arg3-D4*	This study
MCW9276	*h⁺ ade6Δ::RTS1*-IO-*LEU2* (12.4 kb from *RTS1*-IO-*LEU2*) int::*Alu*Sx1/*ura4+*/pUC8/*his3+*/*Alu*Sp/*kanMX6 oriIII-1253Δ::natMX4 ura4-D18 leu1-32 his3-D1 arg3-D4*	This study
MCW9278	*h⁺ ade6Δ::RTS1*-AO-*LEU2* (12.4 kb from *RTS1*-AO-*LEU2*) int::*Alu*Sx1/*ura4+*/pUC8/*his3+*/*Alu*Sp/*kanMX6 oriIII-1253Δ::natMX4 ura4-D18 leu1-32 his3-D1 arg3-D4*	This study
MCW10086	*h⁻ rad52Δ::rad52*-R45A-*kanMX6 ade6Δ::RTS1*-IO-*LEU2* (12.4 kb from *RTS1*-IO-*LEU2*) int::*Alu*Sx1/*ura4+*/pUC8/*his3+*/*Alu*Sp/*hphMX4 oriIII-1253Δ::natMX4 ura4-D18 leu1-32 his3-D1 arg3-D4*	This study
MCW10083	*h⁺ rad52Δ::rad52*-R45A-*kanMX6 ade6Δ::RTS1*-AO-*LEU2* (12.4 kb from *RTS1*-AO-*LEU2*) int::*Alu*Sx1/*ura4+*/pUC8/*his3+*/*Alu*Sp/*hphMX4 oriIII-1253Δ::natMX4 ura4-D18 leu1-32 his3-D1 arg3-D4*	This study
MCW10084	*h⁻ rad52Δ::rad52*-R45A-*kanMX6 ade6Δ::RTS1*-AO-*LEU2* (12.4 kb from *RTS1*-AO-*LEU2*) int::*Alu*Sx1/*ura4+*/pUC8/*his3+*/*Alu*Sp/*hphMX4 oriIII-1253Δ::natMX4 ura4-D18 leu1-32 his3-D1 arg3-D4*	This study
FO652	*h⁻ ura4-D18 leu1-32 his3-D1 arg3-D4*	Lab strain
MCW9093	*h⁺ ade6Δ::RTS1*-IO-*hphMX4* (12.4 kb from *ade6*) int::*ade6+*/*kanMX6 oriIII-1253Δ::natMX4 ura4-D18 leu1-32 his3-D1 arg3-D4*	This study
MCW9094	*h⁺ ade6Δ::RTS1*-AO-*hphMX4* (12.4 kb from *ade6*) int::*ade6+*/*kanMX6 oriIII-1253Δ::natMX4 ura4-D18 leu1-32 his3-D1 arg3-D4*	This study
MCW9105	*h⁺ ade6Δ::RTS1*-IO- *hphMX4* (12.4 kb from *RTS1*-IO- *hphMX4*) int::*Alu*Sx1/*ura4+*/pUC8/*his3+*/*Alu*Sp/*kanMX6 oriIII-1253Δ::natMX4 ura4-D18 leu1-32 his3-D1 arg3-D4*	This study
MCW9109	*h⁺ ade6Δ::RTS1*-AO- *hphMX4* (12.4 kb from *RTS1*-AO- *hphMX4*) int::*Alu*Sx1/*ura4+*/pUC8/*his3+*/*Alu*Sp/*kanMX6 oriIII-1253Δ::natMX4 ura4-D18 leu1-32 his3-D1 arg3-D4*	This study
