# Supplementary material for: Rad52’s DNA annealing activity drives template switching associated with restarted DNA replication
Source: Nat Commun. 2022 Nov 26;13:7293. doi: 10.1038/s41467-022-35060-4 (PMC9701231; doi:10.1038/s41467-022-35060-4)
Supplement: Supplementary file 3 — Description of Additional Supplementary Files [file 41467_2022_35060_MOESM3_ESM.pdf]

Description of Additional Supplementary Files

File Name: Supplementary Data 1
Description: Details of the statistical analysis of the data in Figures 2 – 6, Supplementary Figures 2 – 3 and Supplementary Tables 1 – 2.
