# Supplementary material for: Rad52’s DNA annealing activity drives template switching associated with restarted DNA replication
Source: Nat Commun. 2022 Nov 26;13:7293. doi: 10.1038/s41467-022-35060-4 (PMC9701231; doi:10.1038/s41467-022-35060-4)
Supplement: Supplementary file 6 — Source Data [file 41467_2022_35060_MOESM6_ESM.zip › Source Data/Source data (western blots).pdf]

Rad52's DNA annealing activity drives template switching associated with restarted DNA replication

Anastasiya Kishkevich, Sanjeeta Tamang, Michael O. Nguyen, Judith Oehler,
Elena Bulmaga, Christos Andreadis, Carl A. Morrow, Fekret Osman
and Matthew C. Whitby

Department of Biochemistry, University of Oxford, South Parks Road, Oxford,
OX1 3QU UK

SOURCE DATA

Figure 4b, and Supplementary Figures 2a and 3b

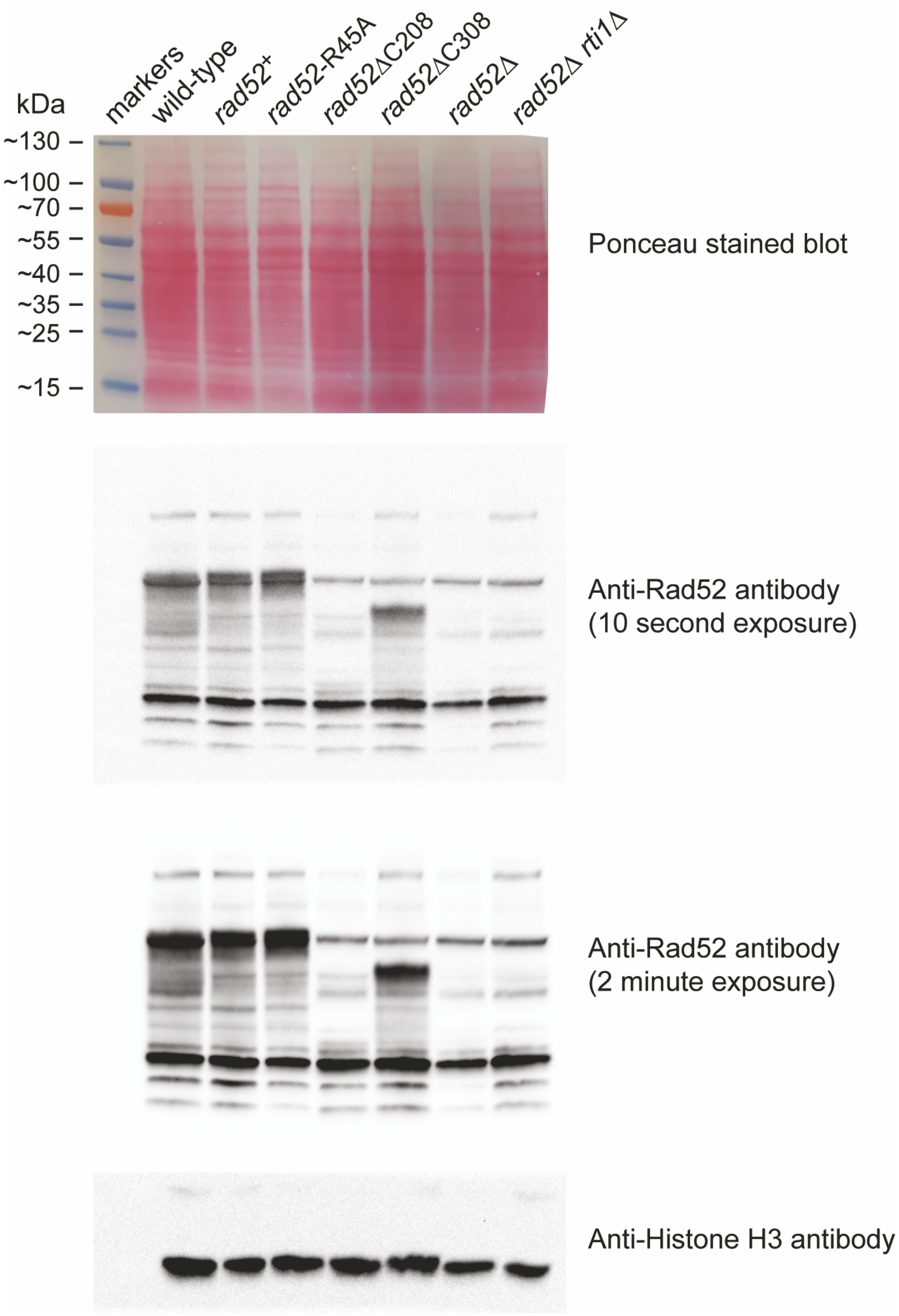

Source data for Figure 4b.

The membrane was stained with Ponceau and then probed with Anti-Rad52 antibody. After detection of Rad52, the membrane was stripped and its bottom portion probed with Anti-Histone H3 antibody. Chemiluminescent signals were detected by a ChemiDoc XRS+ system (Bio-Rad Laboratories, Inc.) using a range of different exposure times.

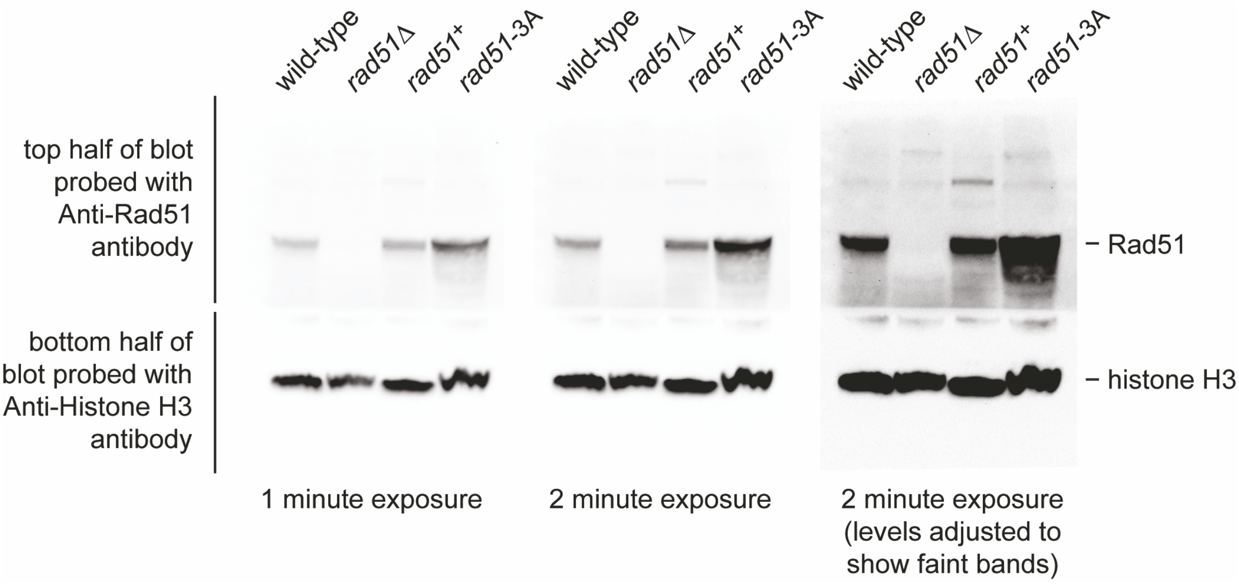

Source data for Supplementary Figure 2a.

The membrane was cut into top and bottom portions with the top portion probed with Anti-Rad51 antibody and the bottom portion probed with Anti-Histone H3 antibody. Chemiluminescent signals were detected by a ChemiDoc XRS+ system (Bio-Rad Laboratories, Inc.) using a range of different exposure times.

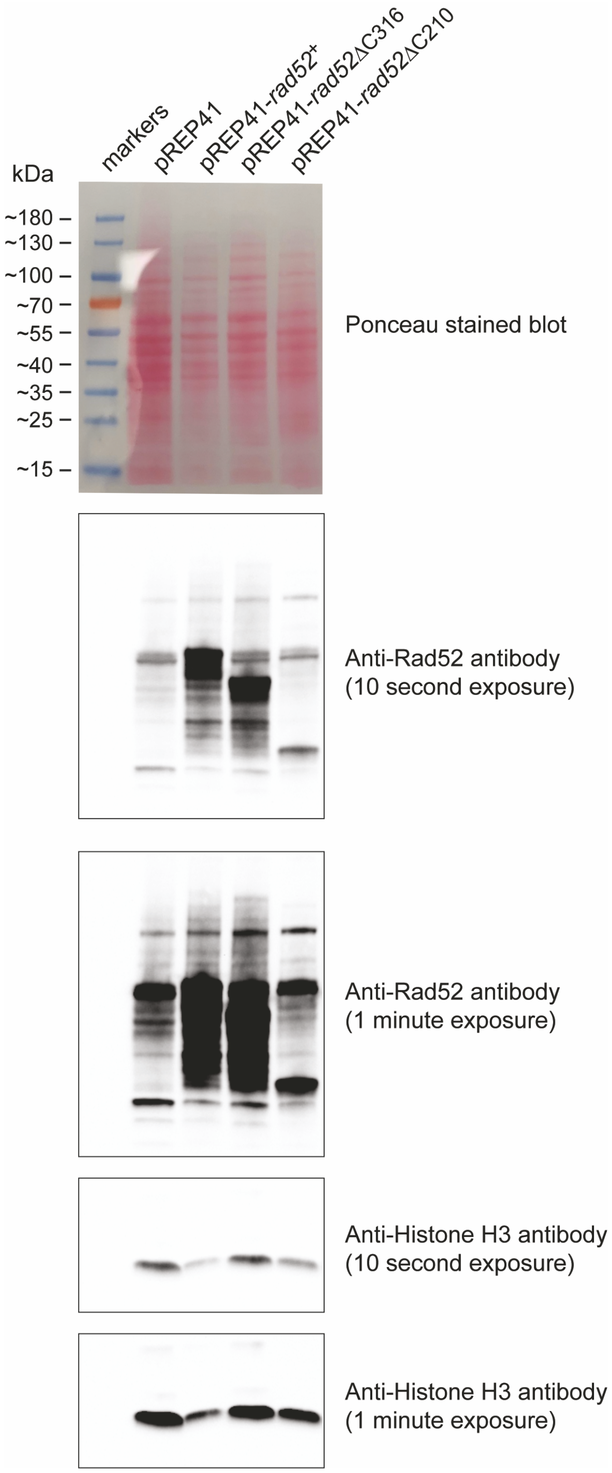

Source data for Supplementary Figure 3b.

The membrane was stained with Ponceau and then probed with Anti-Rad52 antibody. After detection of Rad52, the membrane was stripped and its bottom portion probed with Anti-Histone H3 antibody. Chemiluminescent signals were detected by a ChemiDoc XRS+ system (Bio-Rad Laboratories, Inc.) using a range of different exposure times.
